# Predicting survival rates: the power of prognostic nomograms in distal cholangiocarcinoma

**DOI:** 10.3389/fonc.2025.1478836

**Published:** 2025-01-27

**Authors:** Jiangfeng Hu, Yuping Shi, Lihua Jin, Suhong Yi, Jinsuo Chen, Dadong Wan, Weixin Ye, Jingnan Chen, Yajing Zhang, Yang Jiang, Bensong Duan, Yuwei Dong

**Affiliations:** ^1^ Department of Gastroenterology, Shanghai General Hospital, Shanghai Jiao Tong University School of Medicine, Shanghai, China; ^2^ Department of Nephrology, Tongren Hospital, Shanghai Jiao Tong University School of Medicine, Shanghai, China; ^3^ Department of Gastroenterology, Xinyu People’s Hospital, Xinyu, Jiangxi, China; ^4^ Department of Hepatobiliary and Pancreatic Surgery, Jiangnan University Medical Center, Wuxi, Jiangsu, China; ^5^ Department of Gastroenterology, Fuyang Women & Children’s Hospital, Fuyang, Anhui, China; ^6^ Department of Gastroenterology, Xining Second People’s Hospital, Xining, Qinghai, China; ^7^ Department of Gastroenterology, Shanghai Municipal Hospital of Traditional Chinese Medicine, Shanghai University of Traditional Chinese Medicine, Shanghai, China; ^8^ Department of Neurosurgery, Shanghai Tenth People’s Hospital, Tongji University School of Medicine, Shanghai, China; ^9^ Endoscopy Center, Department of Gastroenterology, Shanghai East Hospital, Tongji University School of Medicine, Shanghai, China

**Keywords:** nomogram, distal cholangiocarcinoma (dCCA), survival, SEER, predict model

## Abstract

**Objective:**

The purpose of this research is to establish a prognostic nomogram for patients with distal cholangiocarcinoma (dCCA).

**Methods:**

We obtained clinical data from 2401 patients diagnosed with distal cholangiocarcinoma (dCCA) between 2010 and 2020 from the Surveillance, Epidemiology, and End Results database. These patients were randomly assigned to either the training or validation group in a ratio of 6:4. 228 patients were enrolled from 9 hospitals in China as the external validation cohort. Univariate and multifactorial Cox regression analyses were conducted to ascertain prognostic factors and prognostic nomograms were developed utilizing LASSO logistic regression analysis. We used the calibration curve, and area under the curve to validate the nomograms. Decision curve analysis was used to evaluate the model and its clinical applicability.

**Results:**

The findings demonstrated that Grade, M stages, Surgery, and Chemotherapy emerged as autonomous prognostic factors for the survival of individuals with dCCA. The developed nomograms exhibited satisfactory accuracy in forecasting 1-year, 3-year, and 5-year survival probabilities. Furthermore, the calibration curves indicated a strong concordance between the anticipated and observed outcomes. The AUC of the nomogram for 1-year, 3-year, 5 year overall survival (OS) predication were 0.809 (95%CI 78.5-83.3), 0.79 (95%CI 75.8-82.2) and 0.761 ((95%CI 72.3-80.0) in the training cohort, 0.79 (95%CI 75.9-82.0), 0.73 (95%CI 68.5-77.5), and 0.732(95%CI 68.0-78.3) in internal test cohort, 0.862 (95%CI 81.7-90.7),0.83 (95%CI 76.4-89.6),and 0.819(95%CI 74.6-89.2) in external test cohort.

**Conclusion:**

The nomograms that have been suggested demonstrate strong predictive capability. These tools can assist medical professionals in assessing the prognosis of patients with dCCA and in devising more accurate treatment strategies for them.

## 1 Introduction

Cholangiocarcinoma is an infrequent yet highly aggressive cancer that originates from the biliary epithelium, with an estimated yearly occurrence of about 1-2 cases per 100,000 people in Western nations (1). The disease is classified based on its anatomical location into intrahepatic, perihilar, and distal cholangiocarcinoma, with distal cholangiocarcinoma accounting for approximately 20-30% of all cholangiocarcinoma cases (2).

Surgical intervention continues to be the primary approach for curative management of distal cholangiocarcinoma and negative surgical margins are the most important predictor of long-term survival (3). Despite curative-intent resection, many patients experience disease recurrence, necessitating the use of adjuvant therapies such as chemotherapy to improve outcomes (4–6).

The TNM staging system, which integrates tumor dimensions (T), lymph node participation (N), and distant metastasis (M), is frequently employed for prognostic purposes in cholangiocarcinoma. However, this system has limitations in predicting individual patient outcomes and guiding treatment decisions (7). In recent times, there have been endeavors to identify additional prognostic factors and develop prediction models to improve the accuracy of survival predictions in patients with cholangiocarcinoma.

Numerous research studies have examined the predictive importance of clinicopathological factors in distal cholangiocarcinoma. These factors encompass direct bilirubin (DBIL), perineural invasion, lymph node metastasis, resection margin status, tumor size, and various others (8–11). Additionally, molecular markers such as KRAS mutations and programmed death-ligand 1 (PD-L1) have been explored as potential prognostic indicators and targets for novel therapies in cholangiocarcinoma (12–14).

In clinical practice, accurate prediction of survival outcomes is crucial for treatment planning and counseling of patients with distal cholangiocarcinoma. Nomograms, which are graphical representations of prediction models, have been increasingly utilized to estimate individualized survival probabilities based on multiple prognostic factors (15). Furthermore, the use of nomograms allows for the integration of diverse prognostic variables and facilitates risk stratification in clinical decision-making (16).

Due to the intricate nature and constraints of existing prognostic instruments for distal cholangiocarcinoma, there is a requirement for comprehensive clinical prediction models that can effectively categorize patients according to their prognosis. In this study, we aim to develop and validate a prediction model for survival outcomes in patients with distal cholangiocarcinoma, incorporating a range of clinicopathological and treatment-related factors. The use of Cox regression and the construction of a nomogram will enable us to create a practical tool for predicting individualized survival probabilities in this patient population.

## 2 Materials and methods

### 2.1 Patient data

The data utilized in this study were acquired from SEER∗Stat (version 8.4.0.1). Inclusion criteria for patients encompassed the following: (1) the year of diagnosis falling from 2010 to 2020, (2) site code: C24.0, and ICD-O- histology/behavior codes: 8140,8160. Patients who satisfied any of the specified criteria were excluded from the study: (1) those with a second primary cancer, and (2) those with a survival period of less than 30 days. Some of the variables were regrouped for the analysis. Patients were re-evaluated based on the staging principles outlined in the eighth edition of the American Joint Committee on Cancer guidelines. Overall survival was defined as the duration between the date of diagnosis and the date of death from any cause or the date of the last follow-up visit.

**Figure 1:**
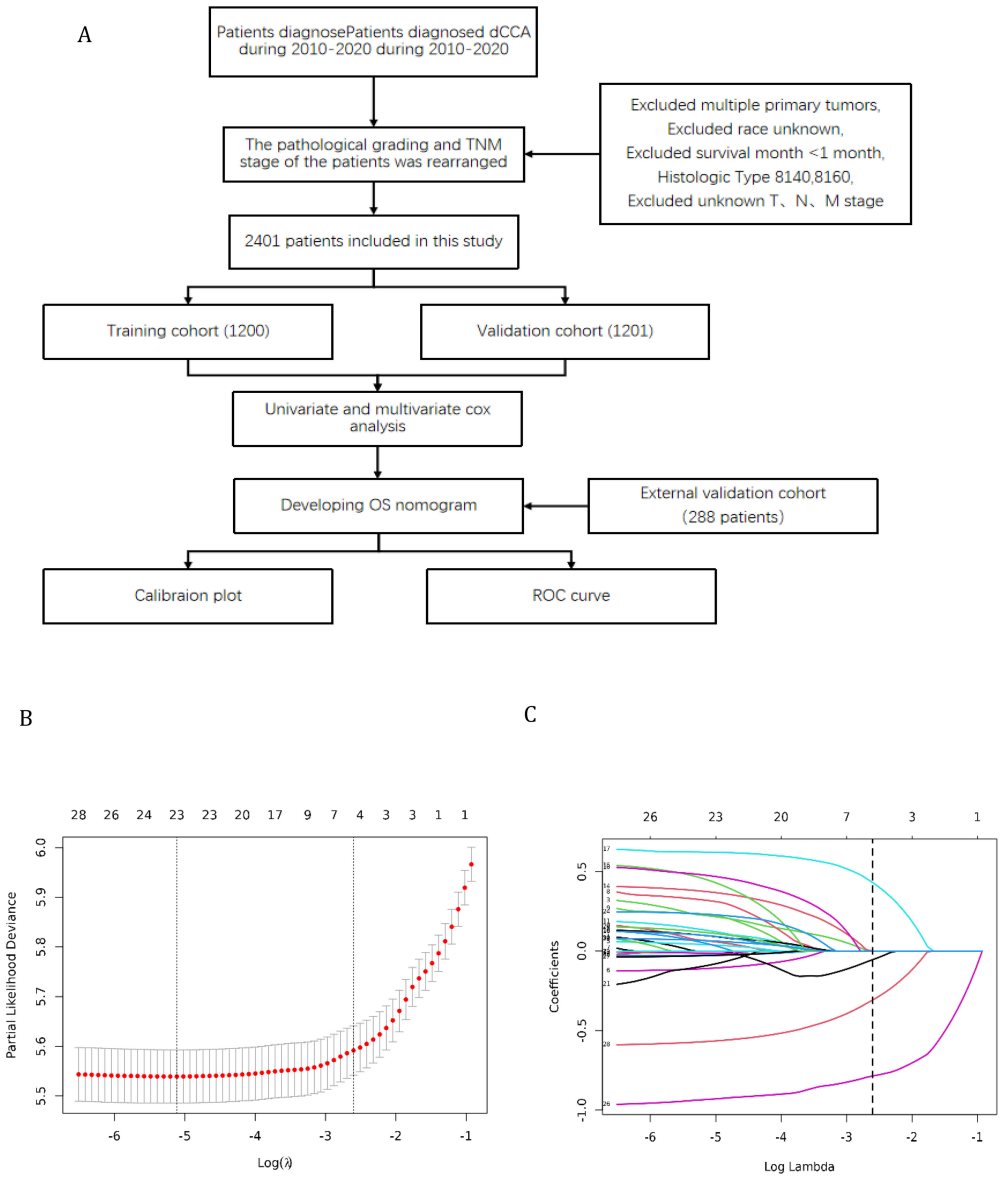
**(A)** Flowchart for selection of research subjects **(B)** Lasso Regression Cross-Validation Plot **(C)** Lasso Regression Coefficient Path Plot.

Furthermore, the external validation set of this study was obtained from 9 hospitals in China, including Shanghai General Hospital, Shanghai Tenth People’s Hospital, Shanghai East Hospital, etc. The flowchart shows the whole process of data screening and data analysis in this study (**Figure 1**).

### 2.2 Data collection

The clinical data of this study population were obtained from publicly available datasets. These data are available here: Surveillance, Epidemiology and End Results (SEER) database. Two different clinicians collated the data from the external validation set. The following demographic and clinical data were obtained in this research: Age, Sex, Race, Grade, TNM stage, Bone metastases, Brain metastases, Liver metastases, Lung metastases, Surgery, Radiation, Chemotherapy, and Marital. The data of outcome and vital status, were also collected.

### 2.3 Statistical analysis and model establishment

The dataset was partitioned into training and validation cohorts randomly, with a 6:4 ratio, and the variables were subsequently analyzed for comparison. In the univariate analysis, categorical variables were analyzed using either the chi-square test or Fisher’s exact test, while continuous variables were examined using either the Student’s t-test or rank-sum test. In the training cohort, multivariate analysis was conducted using the least absolute shrinkage and selection operator (LASSO) Cox regression analysis to ascertain independent risk factors of dCCA and construct a prediction nomogram for the survival probability of dCCA. The nomogram’s performance was assessed by employing the ROC curve and calibration curve. The AUC curve was utilized to measure the discriminant ability, with values ranging from 0.5 (indicating no discriminant ability) to 1 (indicating complete discriminant ability). Additionally, a decision curve analysis (DCA) was conducted to establish the net benefit threshold of prediction. Survival analysis was conducted using the Kaplan-Meier (KM) method. Statistical significance was determined by results with a p-value of less than 0.05. All statistical analyses were carried out using the R software (version 4.2.2). The KM survival and ROC analysis was conducted with the “survival” and “timeROC” packages. The “riskRegression” packages were applied to generate the calibration curve.

## 3 Results

### 3.1 Patient characteristics

In our study examining predictive factors across different cohorts, we analyzed baseline demographic and clinical characteristics to gauge their significance in the research (**Table 1**). Shapiro-Wilk normality test was performed and statistical methods were selected according to the results. The age distribution across cohorts showed a statistically significant difference (p=0.029), with variations noted in the ≥75 age group, ranging from 38.2% in the Training Cohort to 41.7% in the External Test Cohort. Sex distribution displayed a trend towards significance (p=0.069), with the proportion of females ranging from 41.5% in the Internal Test Cohort to 49.0% in the External Test Cohort. A significant difference in race distribution was observed (p<0.001), notably in the Asian subgroup, constituting 100.0% of the External Test Cohort. Moreover, grade distribution demonstrated heterogeneity, with Grade II proportions varying from 21.3% in the Training Cohort to 33.0% in the External Test Cohort (p<0.001). Regarding cancer staging, T and N categories did not show significant differences, while M, reflecting metastasis, exhibited variation (p=0.006), notably in the External Test Cohort. The presence of metastases in different organs, such as bone, brain, liver, and lung, notably showed varied distributions across cohorts, with statistically significant differences noted in liver metastases (p=0.031) and brain metastases (p=0.30). Treatment modalities, including surgery, radiation, and chemotherapy, demonstrated no significant disparities across cohorts, suggesting consistent management strategies. Additionally, marital status distribution exhibited no significant differences, with most individuals being married. Our comprehensive analysis of these baseline characteristics sheds light on the varied profiles within the cohorts and may inform predictive modeling and clinical decision-making in similar contexts.

**Table 1:** Demographic and clinical characteristics of patients across different cohors.

Characteristic	Cohort			p-value^2^
	Training Cohort, N = 1,441^1^	Internal Test Cohort, N = 960^1^	External Test Cohort, N = 288^1^	
Age				0.029
<55	140 (9.7%)	93 (9.7%)	45 (15.6%)	
55-64	315 (21.9%)	210 (21.9%)	49 (17.0%)	
65-74	436 (30.3%)	280 (29.2%)	74 (25.7%)	
≥75	550 (38.2%)	377 (39.3%)	120 (41.7%)	
Sex				0.069
Female	637 (44.2%)	398 (41.5%)	141 (49.0%)	
Male	804 (55.8%)	562 (58.5%)	147 (51.0%)	
Race				<0.001
White	1,066 (74.0%)	738 (76.9%)	0 (0.0%)	
Black	134 (9.3%)	77 (8.0%)	0 (0.0%)	
Asian	241 (16.7%)	145 (15.1%)	288 (100.0%)	
Grade				<0.001
Grade I	56 (3.9%)	64 (6.7%)	17 (5.9%)	
Grade II	307 (21.3%)	208 (21.7%)	95 (33.0%)	
Grade III-IV	270 (18.7%)	156 (16.3%)	86 (29.9%)	
Unknown	808 (56.1%)	532 (55.4%)	90 (31.3%)	
T				0.198
T1	213 (14.8%)	153 (15.9%)	47 (16.3%)	
T2	126 (8.7%)	89 (9.3%)	39 (13.5%)	
T3	556 (38.6%)	348 (36.3%)	107 (37.2%)	
T4	144 (10.0%)	85 (8.9%)	21 (7.3%)	
TX	402 (27.9%)	285 (29.7%)	74 (25.7%)	
N				0.355
N0	789 (54.8%)	562 (58.5%)	151 (52.4%)	
N1	360 (25.0%)	222 (23.1%)	83 (28.8%)	
N2	49 (3.4%)	33 (3.4%)	10 (3.5%)	
NX	243 (16.9%)	143 (14.9%)	44 (15.3%)	
M				0.006
M0	1,101 (76.4%)	760 (79.2%)	202 (70.1%)	
M1	340 (23.6%)	200 (20.8%)	86 (29.9%)	
Bone metastases				0.403
No	1,355 (94.0%)	919 (95.7%)	273 (94.8%)	
Yes	31 (2.2%)	12 (1.3%)	6 (2.1%)	
Unknown	55 (3.8%)	29 (3.0%)	9 (3.1%)	
Brain metastases				0.030
No	1,382 (95.9%)	930 (96.9%)	277 (96.2%)	
Yes	0 (0.0%)	0 (0.0%)	2 (0.7%)	
Unknown	59 (4.1%)	30 (3.1%)	9 (3.1%)	
Liver metastases				0.031
No	1,164 (80.8%)	796 (82.9%)	219 (76.0%)	
Yes	220 (15.3%)	131 (13.6%)	61 (21.2%)	
Unknown	57 (4.0%)	33 (3.4%)	8 (2.8%)	
Lung metastases				0.697
No	1,334 (92.6%)	901 (93.9%)	266 (92.4%)	
Yes	46 (3.2%)	28 (2.9%)	11 (3.8%)	
Unknown	61 (4.2%)	31 (3.2%)	11 (3.8%)	
Surgery				0.975
No/Unknown	806 (55.9%)	540 (56.3%)	160 (55.6%)	
Yes	635 (44.1%)	420 (43.8%)	128 (44.4%)	
Radiation				0.644
No/Unknown	1,146 (79.5%)	766 (79.8%)	236 (81.9%)	
Yes	295 (20.5%)	194 (20.2%)	52 (18.1%)	
Marital				0.658
Married	804 (55.8%)	551 (57.4%)	166 (57.6%)	
Single	194 (13.5%)	123 (12.8%)	44 (15.3%)	
Other	443 (30.7%)	286 (29.8%)	78 (27.1%)	
Chemotherapy				0.160
No/Unknown	707 (49.1%)	500 (52.1%)	156 (54.2%)	
Yes	734 (50.9%)	460 (47.9%)	132 (45.8%)	

^1^n (%).

^2^Pearson's Chi-squared test; Fisher's exact test.

### 3.2 The results of univariate cox regression

Firstly, a univariate analysis was employed to conduct an initial screening of the variables included in the study. A significance level of P < 0.05 was utilized. The findings of the univariate regression analysis are detailed in **Table 2**. It was observed that variables such as Age, Sex, Grade, T stage, N stage, M stage, Surgery, Chemotherapy, Radiotherapy, Bone metastasis, Brain metastasis, Lung metastasis, Liver metastasis, and Marital status may potentially serve as independent risk factors for overall survival.

**Table 2:** Results of univariate cox regression.

Characteristic	N	Event N	HR^1^	95% CI^1^	p-value
Age					
<55	140	93	—	—	
55-64	315	220	1.02	0.80, 1.30	0.853
65-74	436	314	1.10	0.87, 1.39	0.409
≥75	550	453	1.66	1.33, 2.08	<0.001
Sex					
Female	637	510	—	—	
Male	804	570	0.83	0.73, 0.93	0.002
Race					
White	1,066	807	—	—	
Black	134	103	1.14	0.93, 1.39	0.224
Asian	241	170	0.91	0.77, 1.07	0.267
Grade					
Grade I	56	34	—	—	
Grade II	307	189	1.05	0.73, 1.52	0.784
Grade III-IV	270	203	1.51	1.05, 2.17	0.027
Unknown	808	654	2.95	2.09, 4.18	<0.001
T					
T1	213	188	—	—	
T2	126	93	0.59	0.46, 0.75	<0.001
T3	556	382	0.74	0.62, 0.88	<0.001
T4	144	93	0.86	0.67, 1.10	0.236
TX	402	324	1.60	1.33, 1.92	<0.001
N					
N0	789	583	—	—	
N1	360	286	0.93	0.81, 1.07	0.309
N2	49	22	0.71	0.46, 1.08	0.110
NX	243	189	1.81	1.53, 2.14	<0.001
M					
M0	1,101	791	—	—	
M1	340	289	2.42	2.10, 2.78	<0.001
Bone metastases					
No	1,355	1,000	—	—	
Yes	31	28	3.74	2.56, 5.47	<0.001
Unknown	55	52	1.72	1.30, 2.27	<0.001
Brain metastases					
No	1,382	1,024	—	—	
Yes	0	0			
Unknown	59	56	1.73	1.32, 2.27	<0.001
Liver metastases					
No	1,164	838	—	—	
Yes	220	188	2.37	2.02, 2.79	<0.001
Unknown	57	54	1.89	1.44, 2.50	<0.001
Lung metastases					
No	1,334	983	—	—	
Yes	46	39	2.59	1.88, 3.58	<0.001
Unknown	61	58	1.69	1.30, 2.21	<0.001
Surgery					
No/Unknown	806	692	—	—	
Yes	635	388	0.30	0.27, 0.34	<0.001
Radiation					
No/Unknown	1,146	876	—	—	
Yes	295	204	0.59	0.51, 0.69	<0.001
Chemotherapy					
No/Unknown	707	585	—	—	
Yes	734	495	0.55	0.49, 0.62	<0.001
Marital					
Married	804	578	—	—	
Single	194	146	1.35	1.13, 1.62	0.001
Other	443	356	1.48	1.30, 1.70	<0.001

^1^HR, Hazard Ratio; CI, Confidence Interval.

### 3.3 Multivariate analysis of risk factors for dCCA

**Figure 2:**
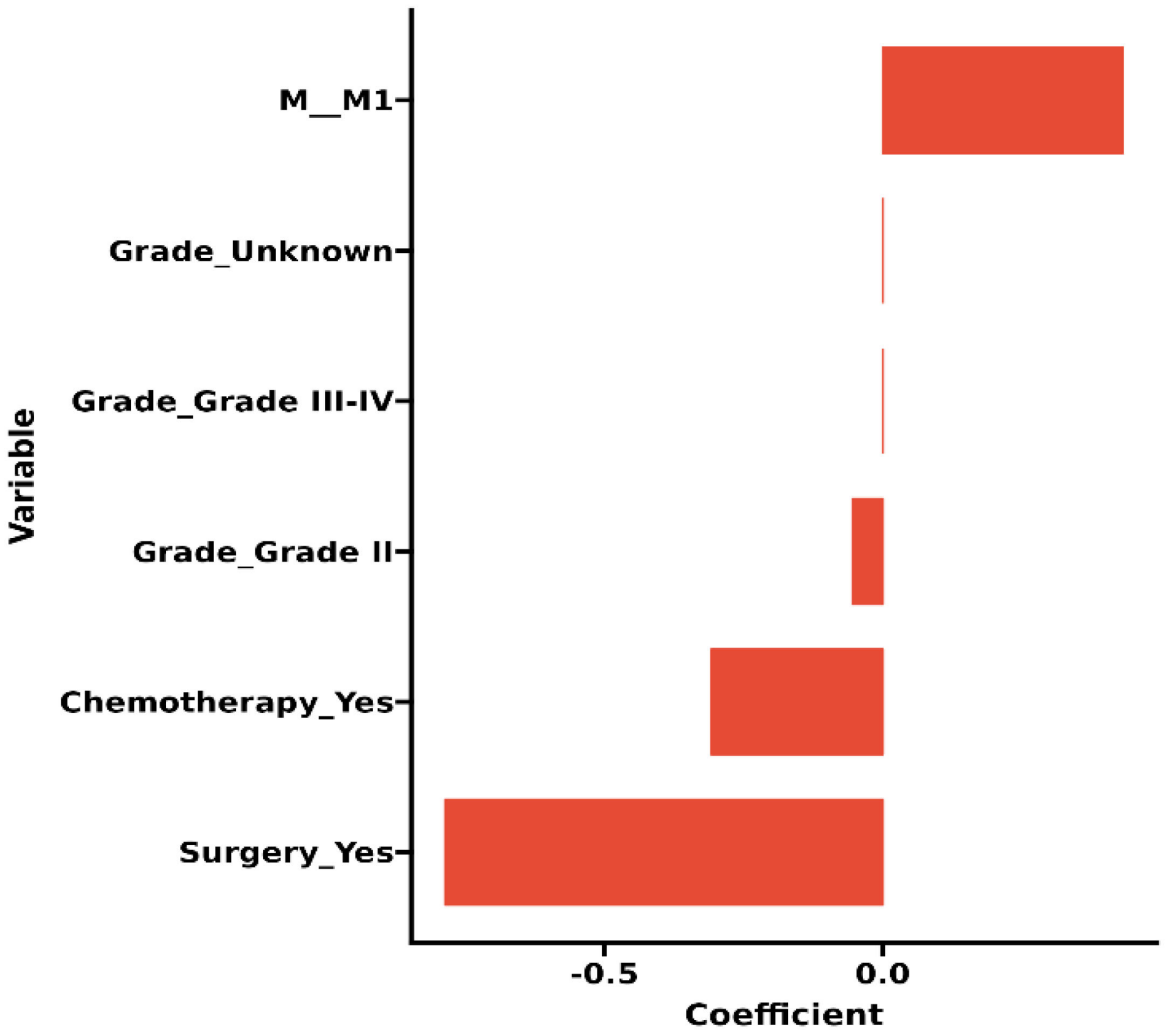
Histogram of the coefficients of the selected features.

The original model included the candidate predictors of Age, Sex, Grade, T stage, N stage, M stage, Surgery, Chemotherapy, Radiotherapy, Bone metastasis, Brain metastasis, Lung metastasis, Liver metastasis, and Marital status. The training cohort was subjected to LASSO regression analysis to determine the significant variables. **Figure 1B** depicts the Cross-Validation Plot for Lasso Regression, with the optimal logλ indicated by the intersection of the red dotted vertical line, denoting the minimum value for the LASSO regression model. The two dotted lines represent one standard deviation from the minimum value. **Figure 1C** illustrates the LASSO coefficient profiles of the potential factors. Each curve corresponds to a coefficient, with the x-axis representing the regularization penalty parameter. As λ changes, a coefficient that becomes non-zero enters the LASSO regression model. The resultant model includes four potential predictors: Grade, M stage, Surgery, and Chemotherapy. The study revealed that all four variables were individually able to predict the survival of dCCA. The details of the selected features are shown in **Figure 2**. According to **Table 3**, patients with Grade III-IV exhibited poorer survival rates in comparison to those with Grade I (HR=1.80, 95% CI 1.25-2.59, p<0.05).Furthermore, M stage (M1 vs M0, HR=2.10, 95% CI 1.80-2.44, p<0.001), Surgery (Yes vs No/unknown, HR=0.39, 95%CI 0.33-0.47, p<0.001), Chemotherapy (Yes vs No/unknown, HR=0.55, 95%CI 0.49-0.63, p<0.001) were identified as independent risk factors for predicting survival. Additionally, the survival outcomes for patients with distal dCCA in relation to Grade, Surgery, Chemotherapy, and M stage were illustrated in **Supplementary Figures** for the training cohort, internal cohort, and validation cohort.

**Table 3:** Results of multivariate cox regression for training cohort.

Characteristic	N	Event N	HR^1^	95% CI^1^	p-value
Grade					
Grade I	56	34	—	—	
Grade II	307	189	1.25	0.87, 1.80	0.236
Grade III-IV	270	203	1.80	1.25, 2.59	0.002
Unknown	808	654	1.49	1.04, 2.14	0.031
M					
M0	1,101	791	—	—	
M1	340	289	2.10	1.80, 2.44	<0.001
Surgery					
No/Unknown	806	692	—	—	
Yes	635	388	0.39	0.33, 0.47	<0.001
Chemotherapy					
No/Unknown	707	585	—	—	
Yes	734	495	0.55	0.49, 0.63	<0.001

^1^HR, Hazard Ratio; CI, Confidence Interval.

### 3.4 Development of a prediction model for predicting dCCA

**Figure 3:**
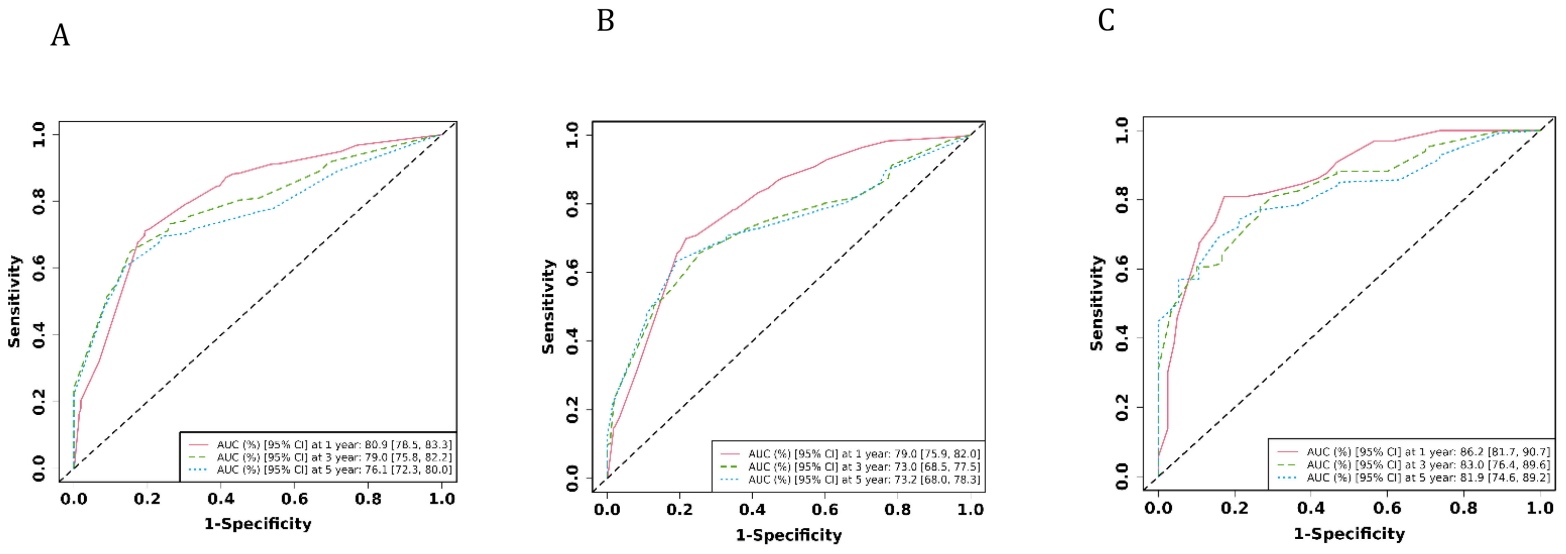
The AUCs of the model in the different cohorts were shown. **(A)** ROC curves of the nomogram prediction model in training cohort. **(B)** ROC curves of the nomogram prediction model in internal test cohort. **(C)** ROC curves of the nomogram prediction model in external test cohort.

**Figure 4:**
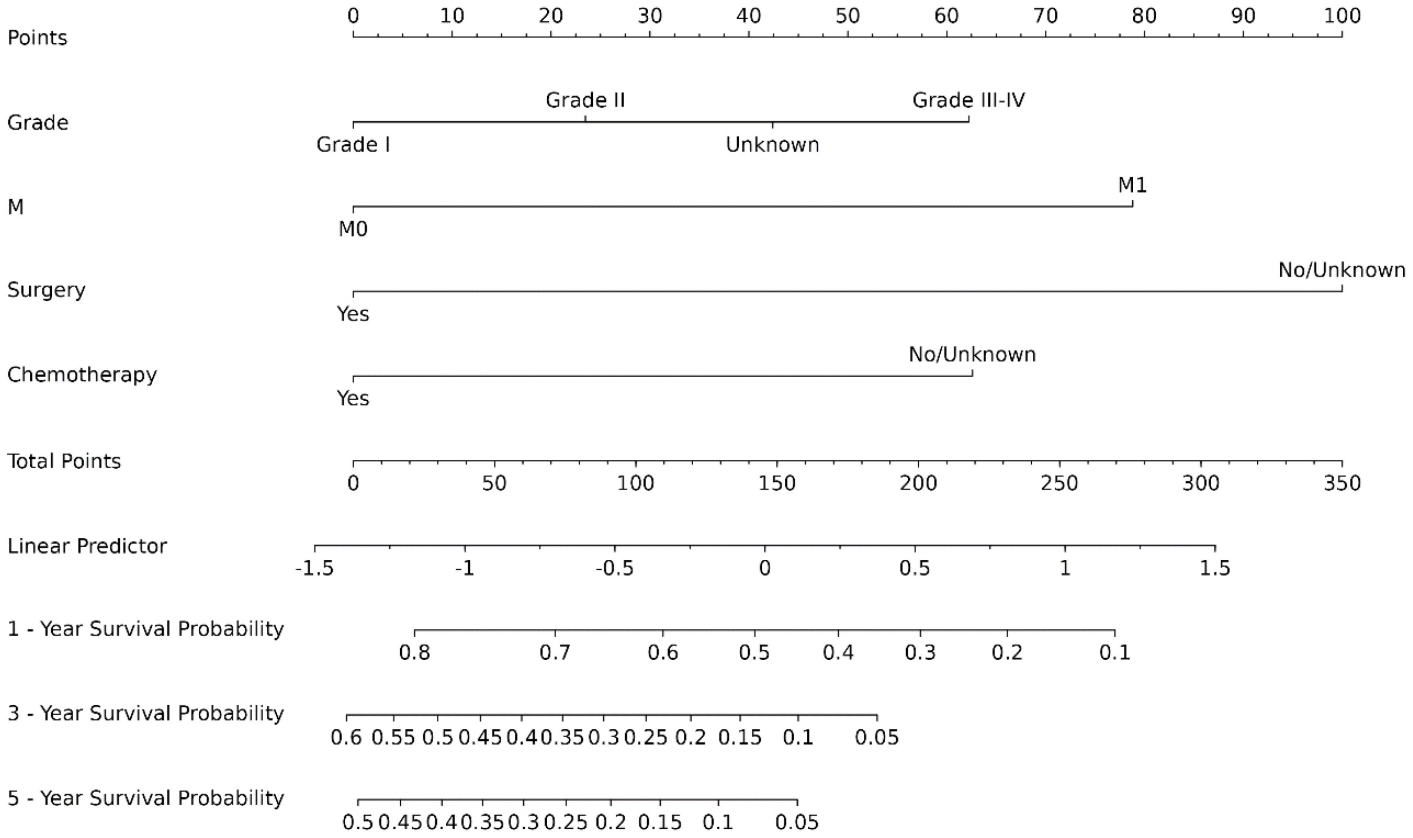
Nomogram prediction model of dCCA.

**Figure 5:**
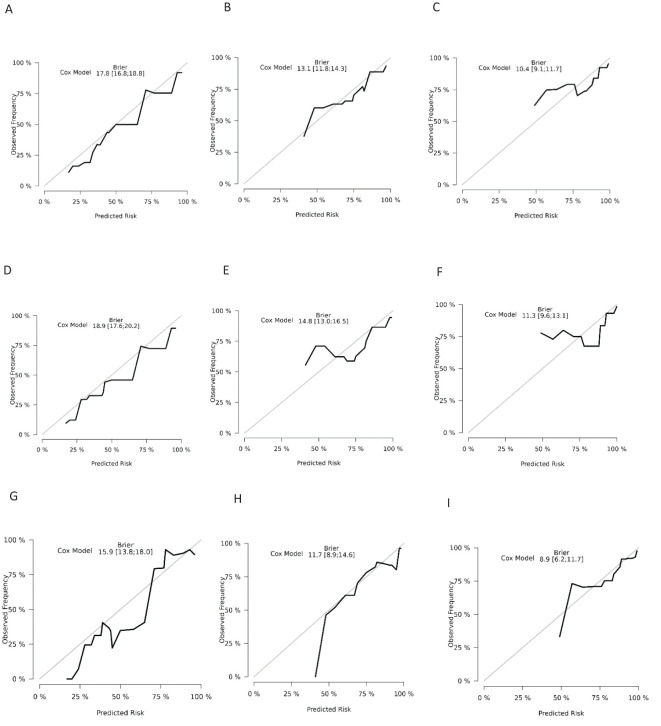
Calibration curves for overall survival nomogram: **(A)** 1 year survival probability in training cohort **(B)** 3 year survival probability in training cohort C.5 year survival probability in training cohort **(D)** 1 year survival probability in validation cohort **(E)** 3 year survival probability in validation cohort **(F)** 5 year survival probability in validation cohort **(G)** 1 year survival probability in external cohort **(H)** 3 year survival probability in external cohort **(I)** 5 year survival probability in external cohort.

The ROC analysis of the aforementioned variables yielded area under the AUC curve values exceeding 0.5, as depicted in **Figure 3**. Consequently, these variables were integrated into the construction of a nomogram, as illustrated in **Figure 4**. The AUC measurements of this model for the training and testing groups were presented. The AUC of the nomogram for 1-year, 3-year, 5 year overall survival (OS) predication were 0.809 (95%CI 78.5-83.3), 0.79 (95%CI 75.8-82.2) and 0.761 ((95%CI 72.3-80.0) in the training cohort, 0.79 (95%CI 75.9-82.0), 0.73 (95%CI 68.5-77.5), and 0.732(95%CI 68.0-78.3) in internal test cohort, 0.862 (95%CI 81.7-90.7),0.83 (95%CI 76.4-89.6),and 0.819(95%CI 74.6-89.2) in external test cohort. The calibration diagrams of the nomogram in the various cohorts are illustrated in the following figures, indicating a robust association between the observed and estimated risk, as depicted in **Figure 5**. These results suggest that the initial nomogram continued to demonstrate effectiveness and accuracy in predicting outcomes when tested on validation sets, closely resembling the ideal curve in the calibration curve.

### 3.5 Decision curve analysis

**Figure 6:**
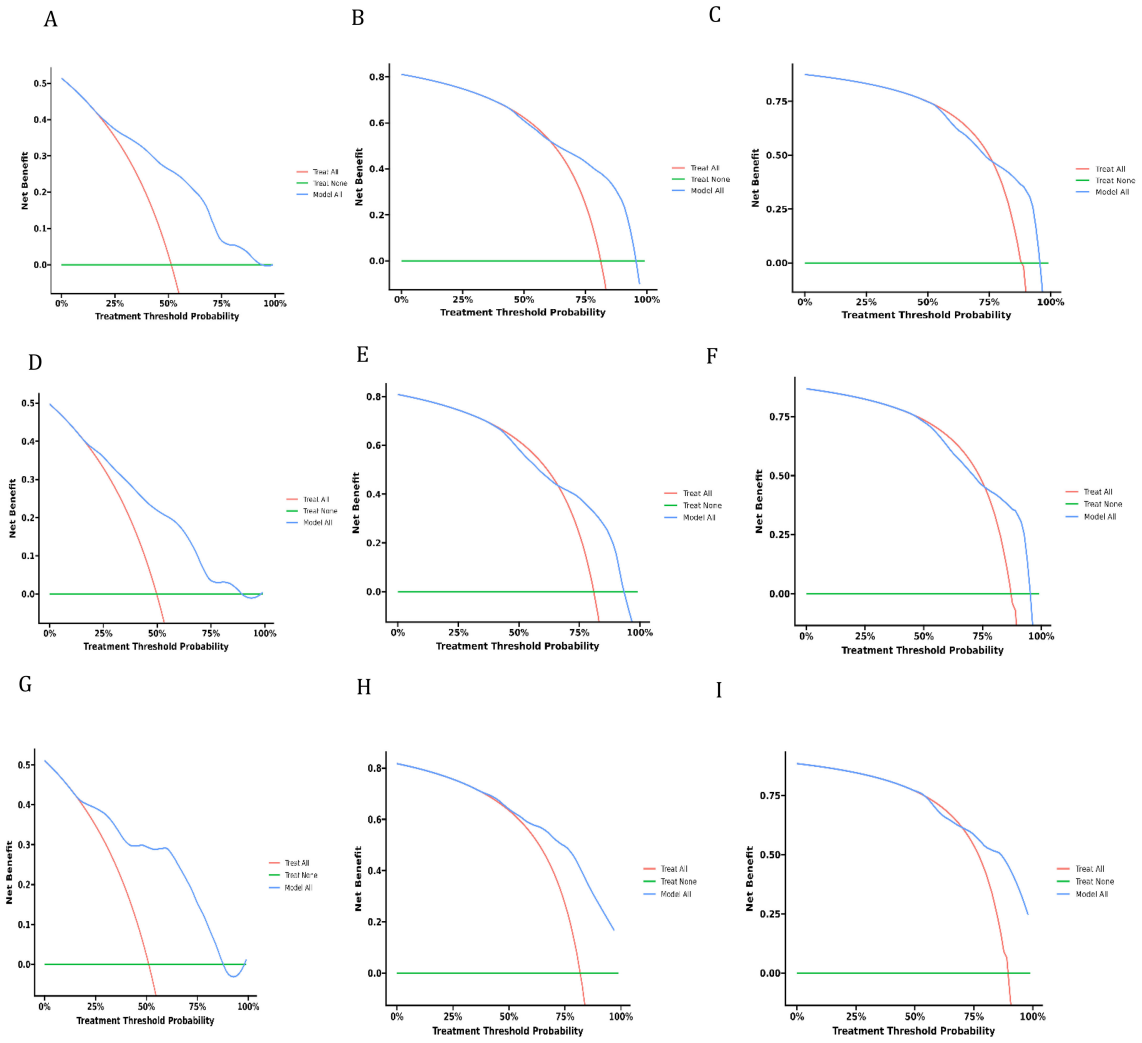
Decision curves of analysis for overall survival nomogram: **(A–C)** in training cohort, **(D–F)**. in validation cohort, **(G–I)** in external cohort.

Decision curve analysis is a novel method that has received approval from many respected academic journals. The net benefit of the prediction models can be computed to assess their clinical utility, a crucial factor for the ultimate implementation of these models in practice. There is not much research in the field of dCCA that uses this method to evaluate the overall benefit of using prediction models because they are relatively new. The DCA curves depicted in **Figure 6** demonstrate the benefits of the nomogram for clinical use, as indicated by this study. It shows that the nomogram provides significant net advantages through its DCA curve.

## 5 Discussion

The majority of patients with dCCA face a grim outlook, as the likelihood of recurrence is high and their life expectancy varies widely, posing challenges in determining survival. Due to the relatively low incidence of dCCA, there is a lack of sufficient sample size to construct an accurate prediction model. We obtained a large number of dCCA patients from the SEER database and several hospitals in China. Extrahepatic cholangiocarcinoma is categorized as either perihilar cholangiocarcinoma (pCCA) or distal cholangiocarcinoma (dCCA). At present, many new studies still focus on extrahepatic cholangiocarcinoma (17–19). However, EASL, ILCA, WHO, and other organizations no longer use the concept of extrahepatic cholangiocarcinoma (20). Because pCCA and dCCA are not only different in location but also have different etiology, risk factors, pathobiology, molecular biology and clinical management (12, 21–23).

Previous studies mainly focused on constructing a nomogram among dCCA patients after surgery. However, the sample size included was too small to affect accuracy, and there was a lack of survival prediction for dCCA patients who did not undergo surgery. Our findings align with the existing literature in some respects. For instance, tumor differentiation has been previously identified as a significant predictor by Ali Belkouz (24). In the current study, we created and tested a nomogram to forecast the survival of dCCA using data from 2689 patients. The main predictors incorporated into the nomogram included Grade, M, Surgery, and Chemotherapy, which were statistically significant in Cox regression analysis. Different from some other studies, there are only four variables in our nomogram. It is suggested that our nomogram is very simple and convenient in actual clinical application, and can quickly help medical workers make judgments.

Although the SEER database does not provide specific surgical methods and specific methods of chemotherapy. However, it seems that surgical methods and specific chemotherapy do not affect the use of our nomogram. dCCA located in bravery manager Vater ampulla and cystic duct and hepatic duct junction, usually for pancreatic duodenal resection. Such as the classical Whipple (the CW) and pylorus-preserving pancreaticoduodenectomy (PPW). There was no significant difference in the prognosis of patients treated with different surgical procedures (25, 26). For many years, chemotherapy has been the mainstay of first-line treatment for advanced biliary tract tumors. According to the NCCN guidelines, the first-line treatment mainly includes gemcitabine alone or gemcitabine combined with cisplatin. In our external validation set, we found that the majority of dCCA patients received consistent chemotherapy regimens. Recently, new therapies for biliary tract cancer, such as immunotherapy, are attracting more and more attention. PD-L1 can be utilized in individuals who have high microsatellite instability or mismatch repair deficiency. However, immunotherapy for biliary tract cancer is currently in the early stages of investigation. The factors used to create the nomogram in this research were easily accessible, and the nomogram developed using the U.S. group still demonstrated high precision when applied to the Asian group. Hence, they exhibit strong fault tolerance and broad applicability, thereby mitigating the impact of variations in medical expertise across different healthcare facilities on the accuracy of predictive outcomes. For dCCA, surgery is the only way to cure patients, but many patients are not suitable for surgical treatment (27–29). To the best of our understanding, this research is the initial attempt to develop a prognostic nomogram that applies to all patients with distal cholangiocarcinoma, regardless of whether they have undergone surgical intervention.

Furthermore, there is a scarcity of treatment approaches tailored to the dCCA population. The utilization of a nomogram has the potential to identify individuals at high risk and facilitate the development of targeted clinical trials, thereby offering valuable insights for the management of dCCA.

## 6 Limitations

Our study has several limitations that should be acknowledged. First, retrospective cohort studies are inevitably subject to selection bias. In order to mitigate this limitation to the greatest extent possible, we incorporated extensive cohorts and partitioned them into a training set and a validation set. The nomogram was developed through randomization and subsequently validated both internally and externally. This approach serves to mitigate potential biases that may arise from retrospective data analysis.

While we have utilized external data from various centers for validation, we aim to confirm the generalizability of our nomograms by replicating our findings in extensive data from additional centers in the future. Furthermore, the incorporation of new predictors or biomarkers may improve the predictive precision of the nomogram, thus necessitating additional research.

## 7 Conclusion

Based on the SEER database, our study identified independent risk factors associated with death in patients with dCCA. Using the identified risk factors, a predictive nomogram was subsequently constructed and rigorously validated in the Chinese dCCA population. The resulting model shows a satisfactory performance. Thus, these nomograms may have profound clinical implications. Their application can largely help medical workers design comprehensive clinical studies, determine personalized treatment strategies, and modify follow-up programs. Taken together, these factors have the potential to significantly improve survival outcomes in patients with dCCA.

## Data Availability

The original contributions presented in the study are included in the article/**Supplementary Material**. Further inquiries can be directed to the corresponding author/s.
