# Supplementary material for: Predicting survival rates: the power of prognostic nomograms in distal cholangiocarcinoma
Source: Front Oncol. 2025 Jan 27;15:1478836. doi: 10.3389/fonc.2025.1478836 (PMC11807801; doi:10.3389/fonc.2025.1478836)
Supplement: Supplementary file 1 [file DataSheet1.docx]

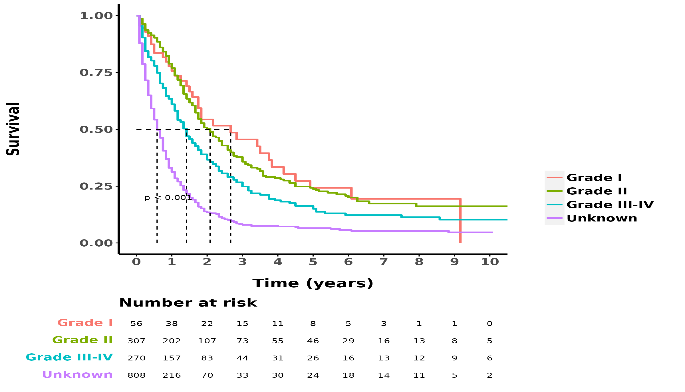

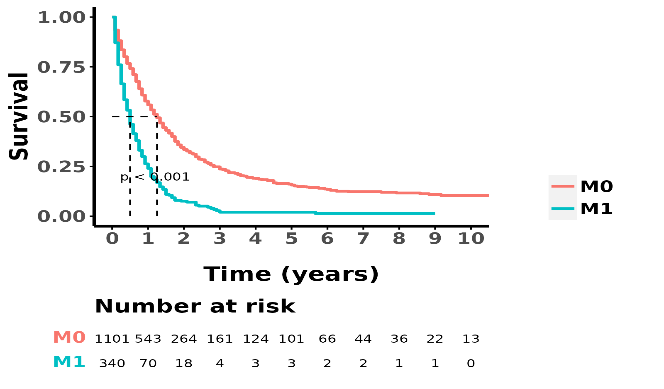


A

**B**


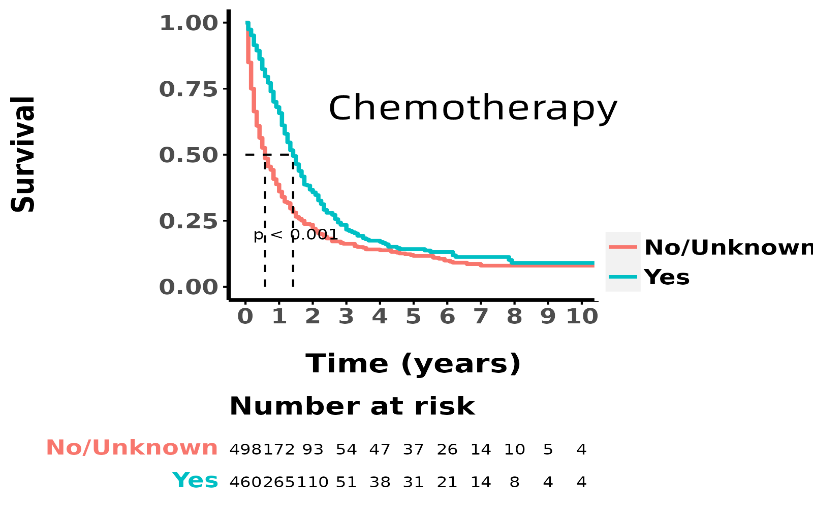

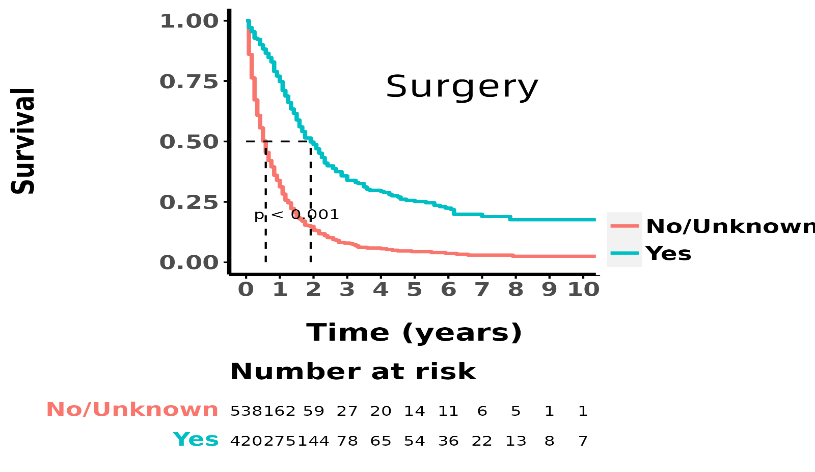

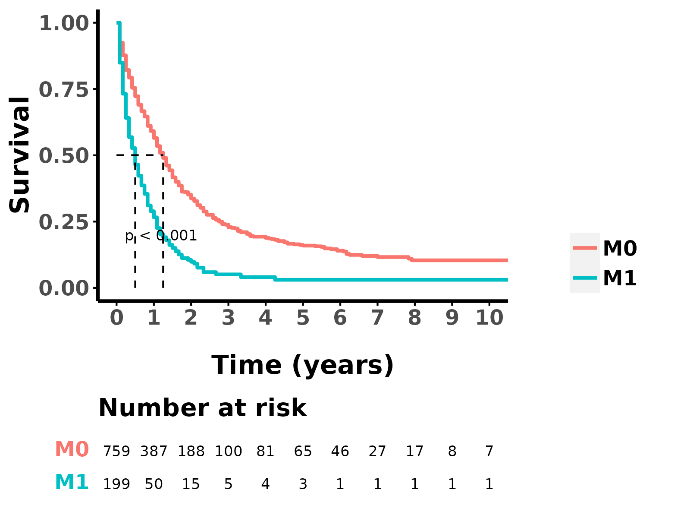

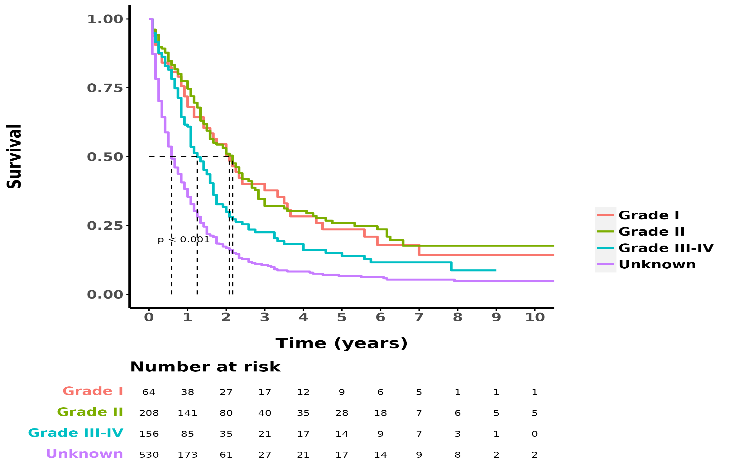

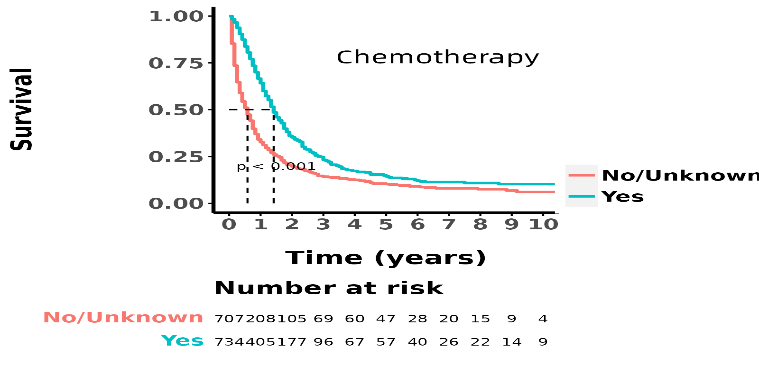

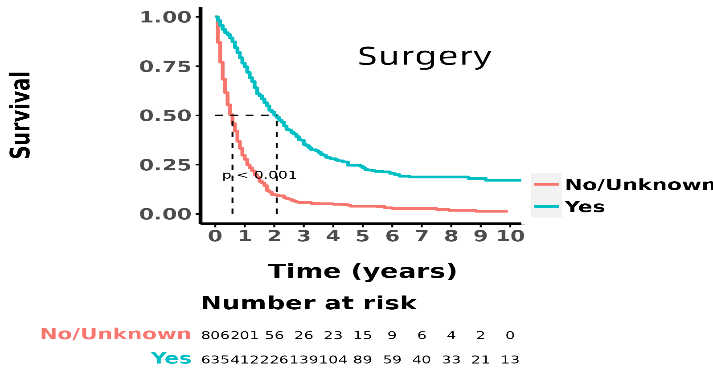


B

Supplementary figure. Kaplan-Meier survival curves for overall survival of dCCA patients (**A**) training cohort (**B**) internal validation cohort; (**C**) external validation cohort


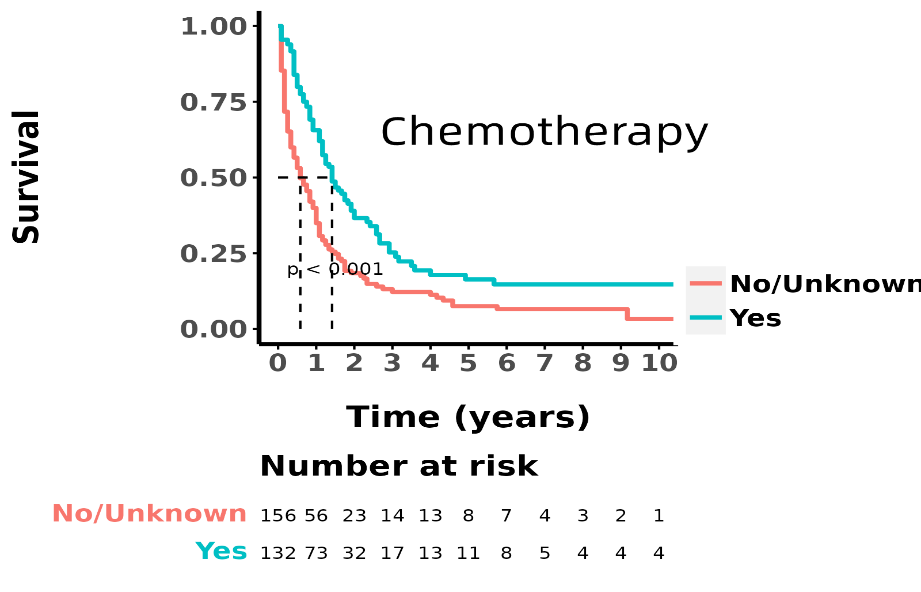

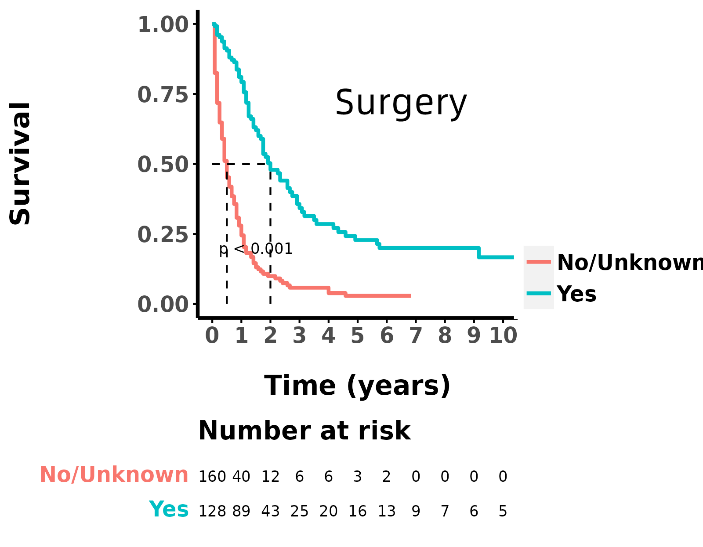

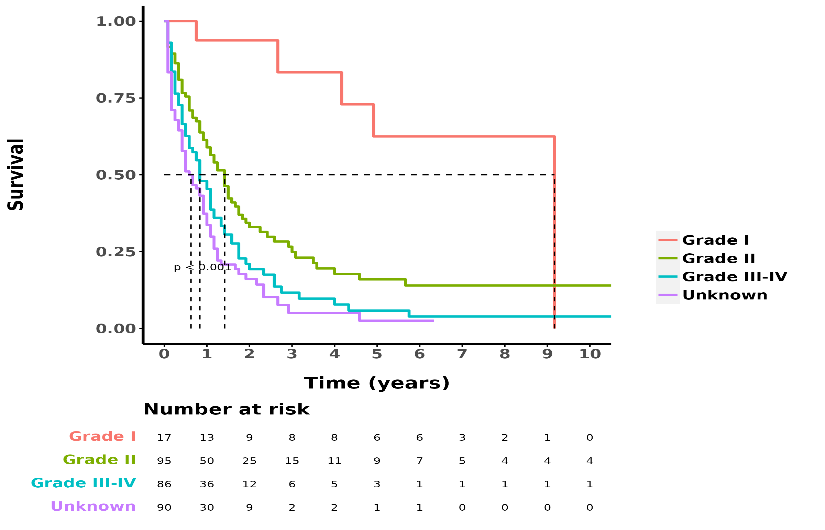

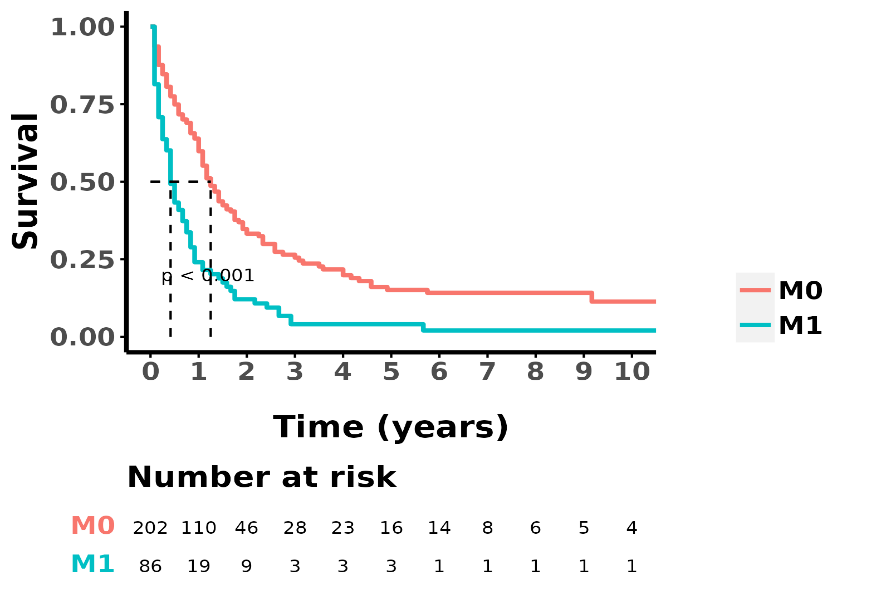


Supplementary figure. Kaplan-Meier survival curves for overall survival of dCCA patients (**A**) training cohort (**B**) internal validation cohort; (**C**) external validation cohort

C
